# ALS-associated missense and nonsense *TBK1* mutations can both cause loss of kinase function

**DOI:** 10.1016/j.neurobiolaging.2018.06.015

**Published:** 2018-11

**Authors:** Martina de Majo, Simon D. Topp, Bradley N. Smith, Agnes L. Nishimura, Han-Jou Chen, Athina Soragia Gkazi, Jack Miller, Chun Hao Wong, Caroline Vance, Frank Baas, Anneloor L.M.A. ten Asbroek, Kevin P. Kenna, Nicola Ticozzi, Alberto Garcia Redondo, Jesús Esteban-Pérez, Cinzia Tiloca, Federico Verde, Stefano Duga, Karen E. Morrison, Pamela J. Shaw, Janine Kirby, Martin R. Turner, Kevin Talbot, Orla Hardiman, Jonathan D. Glass, Jacqueline de Belleroche, Cinzia Gellera, Antonia Ratti, Ammar Al-Chalabi, Robert H. Brown, Vincenzo Silani, John E. Landers, Christopher E. Shaw

**Affiliations:** a United Kingdom Dementia Research Institute, Maurice Wohl Clinical Neuroscience Institute, Institute of Psychiatry, Psychology and Neuroscience, King's College London, London, UK; b Department of Clinical Genetics, Leiden University Medical Center, Leiden, The Netherlands; c Department of Genome analysis, University of Amsterdam, Academic Medical Centre, Amsterdam, The Netherlands; d Department of Neurology, University of Massachusetts Medical School, Worcester, MA; e Department of Neurology and Laboratory of Neuroscience, IRCCS Istituto Auxologico Italiano, Milan, Italy; f Department of Pathophysiology and Transplantation, ‘Dino Ferrari’ Centre, Università degli Studi di Milano, Milan, Italy; g Unidad de ELA, Instituto de Investigación Hospital 12 de Octubre de Madrid, SERMAS, Centro de Investigación Biomédica en Red de Enfermedades Raras (CIBERER U-723), Madrid, Spain; h Department of Biomedical Sciences, Humanitas University, Rozzano-Milan, Italy; i Humanitas Clinical and Research Center, Rozzano-Milan, Italy; j University of Southampton, Southampton General Hospital, UK; k Sheffield Institute for Translational Neuroscience, University of Sheffield, Sheffield, UK; l Nuffield Department of Clinical Neurosciences, University of Oxford, Oxford, UK; m Academic Unit of Neurology, Trinity Biomedical Sciences Institute, Trinity College Dublin, Dublin, Republic of Ireland; n Department of Neurology, Center for Neurodegenerative Disease, Emory University School of Medicine, Atlanta, GA, USA; o Neurogenetics Group, Division of Brain Sciences, Imperial College London, Hammersmith Hospital Campus, London, UK; p Unit of Genetics of Neurodegenerative and Metabolic Diseases, Fondazione IRCCS Istituto Neurologico ‘Carlo Besta’, Milan, Italy

**Keywords:** ALS, TBK1, FTD, WES, Familial ALS

## Abstract

Mutations in TANK binding kinase 1 (TBK1) have been linked to amyotrophic lateral sclerosis. Some *TBK1* variants are nonsense and are predicted to cause disease through haploinsufficiency; however, many other mutations are missense with unknown functional effects. We exome sequenced 699 familial amyotrophic lateral sclerosis patients and identified 16 *TBK1* novel or extremely rare protein-changing variants. We characterized a subset of these: p.G217R, p.R357X, and p.C471Y. Here, we show that the p.R357X and p.G217R both abolish the ability of TBK1 to phosphorylate 2 of its kinase targets, IRF3 and optineurin, and to undergo phosphorylation. They both inhibit binding to optineurin and the p.G217R, within the TBK1 kinase domain, reduces homodimerization, essential for TBK1 activation and function. Finally, we show that the proportion of TBK1 that is active (phosphorylated) is reduced in 5 lymphoblastoid cell lines derived from patients harboring heterozygous missense or in-frame deletion *TBK1* mutations. We conclude that missense mutations in functional domains of TBK1 impair the binding and phosphorylation of its normal targets, implicating a common loss of function mechanism, analogous to truncation mutations.

## 1 Introduction

Amyotrophic lateral sclerosis (ALS) is an adult onset and progressive neurodegenerative disorder that targets the upper and lower motor neurons in the brain and spinal cord. Death usually occurs within 3 to 5 years from the symptom onset, and treatment is largely palliative (Morgan and Orrell, 2016). ALS is often associated with cognitive changes linked to mild frontotemporal dementia (FTD) (Gijselinck et al., 2015), and up to 50% of the FTD cases develop signs of motor neuron disease (van der Zee et al., 2017).

Approximately 10% of ALS cases have a familial history of ALS or FTD (fALS, fALS/FTD) (Tiwari et al., 2005). To date, more than 40 genes have been identified to be associated with ALS through linkage studies, genome-wide association studies, whole exome sequencing, and whole genome sequencing. Four genes account for over 50% of fALS cases: *SOD1, C9ORF72, TARDBP*, and *FUS/TLS* in population of European ancestry, and most other genes are rare, each accounting for ∼1% of the cases (Taylor et al., 2016).

**Fig. 1:**
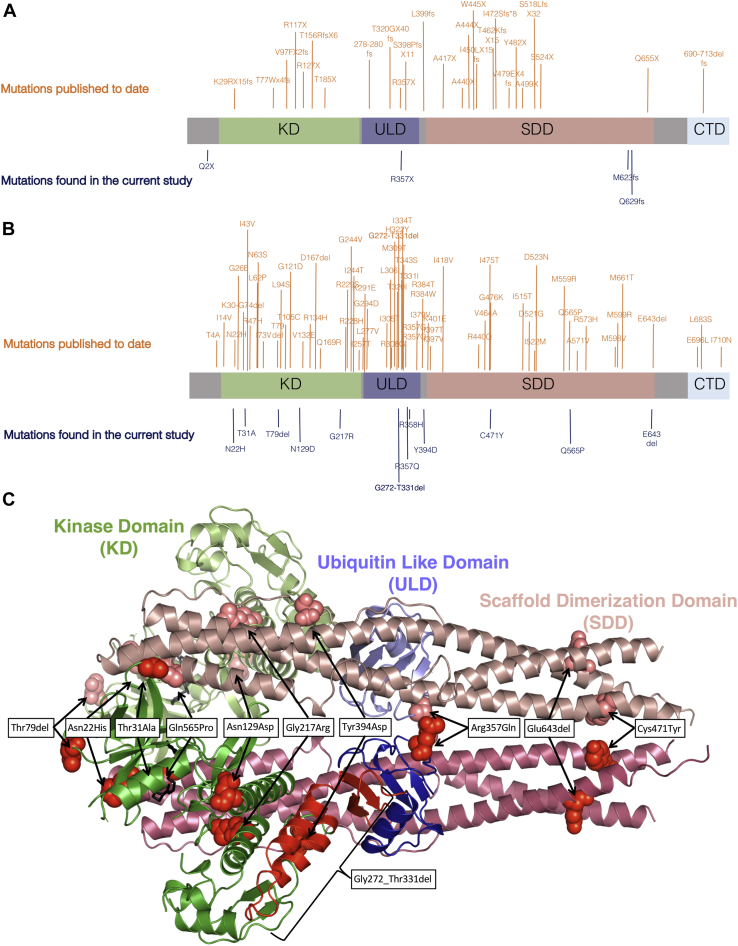
TBK1 mutations identified to date and their location in TBK1 structure. (A, B) Schematic representation of TBK1 protein structure (Tu et al., 2013) showing a map of nonsense (A) and missense (B) variants found in the literature and in our cohort. For more details on the variants found in this study see Table 1. (C) TBK1 homodimer crystal structure (PDB 4IM0) mapping the mutations found in our study excluding premature stop codons and frameshift deletions. Abbreviations: CTD, C-terminal domain (light blue); KD, kinase domain (green); SDD, scaffold dimerization domain (pink); TBK1, TANK binding kinase 1; ULD, ubiquitin-like domain (purple). (For interpretation of the references to color in this figure legend, the reader is referred to the Web version of this article.)

TANK binding kinase 1 (*TBK1*, *NAK*, *T2K*) codes for a protein kinase involved in many pathways including the immune response and autophagy (Weidberg and Elazar, 2011). TBK1 is composed of 4 domains: a kinase domain (KD), responsible for its kinetic activity, an ubiquitin-like domain (ULD), a scaffold dimerization domain (SDD), and a C-terminal domain, involved in TBK1 association with binding partners such as optineurin (OPTN), an important autophagy receptor (Tu et al., 2013) (Fig. 1). TBK1 has been shown to homodimerize through a central axis formed by the 2 SDD domains interacting with each other (Fig. 1C). This structure is stabilized by the ULD and the KD that interact with each other and with the SDD axis, forming a globular head that stabilizes the whole structure (Tu et al., 2013). The interactions between the ULD, the SDD, and their linker region are highly hydrophobic and prevent the homodimer from being dissociated when carrying out its functions. On the other hand, interactions of the KD within this structure are mainly polar (Tu et al., 2013). TBK1 activation has been demonstrated to be a multistep process that begins with the Lys-63-linked polyubiquitination, which is required for Ser172 phosphorylation within the activation loop. This causes a critical change in the protein conformation promoting the active position of the C-helix in the SDD domain and facilitating the final step of homodimerization, essential for mature kinase activity (Ma et al., 2012, Tu et al., 2013).

Mutations in *TBK1* have been recently linked with ALS and FTD by 2 independent whole exome sequencing/whole genome sequencing studies (Cirulli et al., 2015, Freischmidt et al., 2015). Many ALS-linked *TBK1* mutations generate premature stop codons, leading to nonsense-mediated mRNA decay and haploinsufficiency that is predicted to impair autophagy (Freischmidt et al., 2016). However, the pathogenicity and mechanism of missense mutations are unclear (Freischmidt et al., 2016). Here, we describe 16 novel or extremely rare, potentially deleterious variants in *TBK1* and demonstrate that missense mutations can lead to a loss of TBK1 kinase activity by possibly disrupting homodimer formation, phosphorylation of itself and its targets OPTN and interferon regulatory factor 3 (IRF3), implicating a loss of function pathogenic mechanism.

## 2 Material and methods

### 2.1 Patients and DNA samples

All patients and controls gave full patient consent for research purpose. DNA was extracted from 932 patient samples primarily of European ancestry of which 757 were index cases and 175 were affected relatives. All patients had a diagnosis of ALS following revised El Escorial criteria (Brooks et al., 2000) with at least 1 family member affected by ALS and/or FTD. Any sample positive for mutations in known ALS genes (e.g., *SOD1*, *C9orf72*, *TARDBP*, *FUS*, *PFN1*, *UBQLN2*, *OPTN*, *VCP*, and *ANG*) were excluded from further analysis, resulting in a final cohort of 699 probands. Exome sequence data for 102 FALS cases in this cohort were obtained, with permission, from the dbGAP (database of Genotypes and Phenotypes) repository (National Institutes of Health Exome Sequencing of FALS, National Institute of Neurological Disorders and Stroke, phs000101. v4.p1, Traynor).

### 2.2 Exome sequencing and variant analysis

Exomes were captured from the UK samples using the Roche-Nimblegen SeqCap EZ Exome probe library and sequenced on an Illumina HiSeq 2000 producing 100-bp paired-end reads. All other exomes were provided as FASTQ files, captured with a variety of probe sets, and sequenced to produce 50-, 75-, or 100-bp Illumina paired-end reads. Novocraft NovoAlign was used to align the FASTQ files to the hg19 human reference, and variants were called with SAMtools v1.1 mpileup then normalized with bcftools v1.1 norm. Individual variant call files were filtered by the following criteria—DP ≥ 10, QUAL>20, GQ ≥ 50, and MQ ≥ 50—then merged to a single-cohort variant call file. Common ancestry between samples was taken from existing familial annotation where available and also deduced from inheritance by descent analysis in vcftools (Yang et al., 2011) and King (Manichaikul et al., 2010), using only variant positions covered to a depth >10 in >85% of FALS cases, and recoding all missing data to a heterozygous reference genotype (0/0). Functional annotation, pathogenicity predictions, AdaBoost & Random Forest splicing predictions (Jian et al., 2014), and matches to 1000 genomes were added with table_annovar.pl (Wang et al., 2010), whereas all other annotations, including variant frequencies in Exome Sequencing Project (http://evs.gs.washington.edu/EVS), Exome Aggregation Consortium (ExAC, http://exac.broadinstitute.org) and UK10K (www.uk10k.org), were added via custom perl scripts. Variants were removed if they had a carrier frequency of greater than 1 in 20,000 in the non-Finnish European (NFE) subset of ExAC (MAF>0.0025%) or were predicted benign by at least 15 of the 20 pathogenicity prediction algorithms. Synonymous and intronic variants were assessed by NetGene2 and GeneSplicer and excluded if no changes in scores were observed compared with the reference allele at locations matching to known Refseq acceptor or donor splice sites. 5′ and 3′ UTR variants were excluded from consideration in this analysis. To assess the relative abundance of *TBK1* variants in our cohort compared with ExAC, a burden test (Fisher's Exact, 2 tailed) was performed between the number of FALS and ExAC NFE variants remaining after annotation and filtering by the aforementioned criteria.

### 2.3 Plasmid and cloning

HA-tagged *TBK1* wild-type (WT) and FLAG-tagged *OPTN* pCMV3 expression vectors were purchased from Creative Biogene Biotechnology. Single amino acid changes (p.G217R, p.R357X, p.C471Y) were introduced in the HA-tagged *TBK1* WT plasmid by using Q5 Site-Directed Mutagenesis Kit according to the manufacturer's protocol (New England Biolabs). All constructs were verified by Sanger sequencing.

### 2.4 Antibodies

Mouse and rabbit HA-tag monoclonal antibodies were used at a dilution of 1/1000 for Western blot and 1/500 for immunocytochemistry (ICC) (cat no. 2367 and 3724, Cell Signaling Technology). Rabbit anti-TBK1 monoclonal antibody was used at a dilution of 1/1000 (cat no ab40676, Abcam). Rabbit anti-phospho-TBK1 (S172) monoclonal antibody was used at a dilution of 1/500 for Western blot and 1/50 for ICC (cat no. 5483, Cell Signaling Technology). Rabbit anti-IRF3 polyclonal antibody was used at a dilution of 1/200 (cat no. A022993, Bioassay Technology Laboratory). Rabbit anti-phospho-IRF-3 (Ser396) monoclonal antibody was used at a dilution of 1/200 for ICC and Western blot (Cat no. ab76493, Abcam). Mouse anti-DYKDDDDK (FLAG) monoclonal antibody was used at a dilution of 1/3000 (Cat no. TA5001, Origene). Mouse anti–glyceraldehyde 3-phosphate dehydrogenase monoclonal antibody was used at a dilution of 1/6000 (Cat no. G8795, Sigma-Aldrich).

### 2.5 Culture of lymphoblastoid cell lines

Lymphoblastoid cell lines (LCLs) derived from FALS patients and healthy controls were obtained from the European Collection of Authenticated Cell Cultures. LCLs were grown in RPMI media (Gibco; Life Technologies) complemented with 10% Fetal Bovine Serum (Life Technologies), 5% PenStrep (penicillin 100 U/mL and streptomycin 100 U/mL; Life Technologies), and 5% L-glutamine (Life Technologies). These cells grow in suspension and were, therefore, kept in upright T25 flasks (Nunc; Life Technologies) in a water-jacketed 5% CO_2_ incubator.

### 2.6 Culture and transfection of HEK293T cells

HEK293T cells were cultured in Dulbecco's modified Eagle's medium with 10% Fetal Bovine Serum, 5% PenStrep (penicillin 100 U/mL and streptomycin 100 U/mL), 5% glutamine (Life Technologies) in a water-jacketed 5% CO_2_ incubator. For Western blot, native gel and immunoprecipitation (IP) analysis cells were plated in 6-well plates (Life Technologies) and transfected with 1 μg of plasmid DNA, 2 μL of Lipofectamine 2000 (Life Technologies) and 100 μL of Opti-MEM (Life Technologies) per well according to manufacturer's instructions. For immunofluorescence (ICC), HEK293T cells were plated in 24-well plates (Life Technologies) on 13-mm-diameter coverslips (VWR) precoated with poly-D-lysine (Sigma-Aldrich) and transfected with 250 ng of plasmid DNA, 0.5 μL of Lipofectamine 2000 (Life Technologies), and 25 μL of Opti-MEM (Life Technologies) per well, according to manufacturer's instructions. Cells were processed for Western blot, IP, native gel, or ICC after 48 hours of transient transfection.

### 2.7 RNA extraction and RT-PCR

Total RNA was extracted using RNeasy Mini Kit (Qiagen) according to the manufacturer's protocol. The extracted RNA was used as a template for the synthesis of complementary DNA (cDNA) through reverse transcription, using SuperScript III Reverse Transcriptase (Life Technologies) following the manufacturer's protocol. Oligo dT were used to synthesize cDNA. cDNA was amplified using the PCR primers ATGCAGAGCACTTCTAATCATCTGTGGC and CTAAAGACAGTCAACGTTGCGAAG and Sanger sequenced using the sequencing primers TTGAAGGGCCTCGTAGGAAT and TCAGCCATCGTATCCCCTTT.

### 2.8 Immunocytochemistry (ICC)

Forty eight hours after transfection cells were fixed with 4% PFA at room temperature for 15 minutes, permeabilized with 0.2% Triton-X-100 for 30 minutes and blocked with 5% Goat Serum (Sigma) for 1 hour at room temperature. Samples were incubated with primary antibody (anti-HA tag 1/500, anti-pTBK1 1/50, anti-pIRF3 1/200) in 1% Goat Serum overnight at 4 °C. Fluorescent-tagged secondary antibodies (Alexa Fluor 488 Goat IgG Antibody, Alexa Fluor 568 Goat IgG Antibody, 1/500 Life Technologies) were used for fluorescence detection according to manufacturer's instructions. As a negative control samples were incubated with primary antibody only or secondary antibody only (data not shown), DAPI (4′,6-diamidino-2-phenylindole) was used to detect the nuclei. Coverslips were mounted on microscope slides (Thermo Scientific) and imaged using the Leica confocal SP5 microscope (Leica).

### 2.9 Western blot analysis

HEK293T cells were harvested 48 hours after transfection in Phosphate-Buffer Saline (PBS; Severn Biotech LTD) complemented with phosphatase inhibitors (PhoSTOP; Roche) and proteinase inhibitors (COMPLETE; Roche). LCLs were harvested by collecting cells in 15 mL tube (Falcon), centrifuged to form a pellet and resuspended in PBS complemented with phosphatase inhibitors (PhoSTOP; Roche) and proteinase inhibitors (COMPLETE; Roche). Cells were then lysed and processed as previously described (Scotter et al., 2014). Membrane imaging was conducted using goat anti-rabbit and anti-mouse IgG (H+L) DyLight 680 Conjugate (cat. no. 35568 and 35521, Thermo Life Sciences) and an LI-COR Odyssey or using horseradish peroxidase secondary antibodies for mice (Millipore, 12-349) or rabbits (Millipore, 12-348) and developed through an Enhanced Chemiluminescence System using Medical Film Processor SRX-101A (Konica Minolta). Western blot quantification was performed using the image analysis software, ImageJ (http://imagej.nih.gov/ij/(Schindelin et al., 2012)).

### 2.10 Native gel electrophoresis

Cells were harvested in PBS complemented with phosphatase inhibitors and proteinase inhibitors. Samples were then processed using the NativePAGE Novex Bis-Tris Gel System according to manufacturer's protocol. Proteins were transferred on a polyvinylidene difluoride membrane, previously activated in methanol, using the wet transfer system (300 mA, 1 hour). The membrane was then incubated in 8% acetic acid, air-dried, and washed in methanol. A blocking solution of 5% bovine serum albumin was used to block the membrane for 30 minutes prior incubation with 1% bovine serum albumin and primary antibody (anti-HA tag 1/200) at 4 °C overnight. Membrane imaging was conducted using goat anti-rabbit and anti-mouse IgG (H+L) DyLight 680 Conjugate (cat. no. 35568 and 35521, Thermo Life Sciences) and an LI-COR Odyssey.

### 2.11 Cotransfection IP assay

Cells were transfected with *TBK1* WT and mutant plasmids together with *OPTN* WT plasmids. Additional controls untransfected and transfected with either *TBK1* WT or *OPTN* WT only were used. After 48 hours, cells were harvested in IP buffer (50 mM tris [pH 7.4], 150 mM NaCl, 1% Triton-X-100, and 100 mM CaCl_2_ with protease and phosphatase inhibitors). Lysates were partly harvested and diluted in loading buffer complemented with 250 mM 1,4-dithiothreitol (DTT; Thermo Scientific). The lysates were pre-cleaned through incubation with Dynabeads Protein G for IP (Life Technologies) at 4 °C for 2 hours. The beads were then discarded and the lysate was incubated with Dynabeads Protein G and anti-HA tag antibody (1/100) at room temperature for 2 hours. As an additional negative control, 2 samples transfected with *OPTN* WT only or *TBK1* WT only were incubated with beads and no antibody, to reveal any unspecific binding. The beads were separated from the flow-through by magnetic separation and washed with IP buffer 6 times before elution in loading buffer complemented with DTT. Lysates and IP fractions were analyzed by Western blot.

### 2.12 Phosphatase assay

Cells were transfected with *TBK1* WT and mutant plasmids together with *OPTN* WT plasmids. Additional controls including untransfected and transfected with either *TBK1* WT or *OPTN* WT only were used. After 48 hours, transfected cells were harvested in RIPA buffer (50 mM Tris pH 8.0, 150 mM NaCl, 1% NP-40, 0.5% sodium deoxycholate, 0.1% sodium dodecyl sulfate with protease inhibitor) and sonicated for 10 seconds. Six microgram of protein per sample was added to 3 μL of CIP buffer (100 mM NaCl, 50 mM Tris-HCl, 10 mM MgCl_2_, 1 mM dithiothreitol, pH to 7.9 at 25 °C), and 3 μL of alkaline phosphatase (Roche) was added to the phosphatase positive samples, according to the Abcam protein dephosphorylation protocol (http://www.abcam.com/protocols/protein-dephosphorylation-protocol). All the samples were incubated for 30 minutes at 37 °C and ran on NuPAGE Novex 3%–8% Tris-Acetate Midi Protein Gels (Life Technologies). Membrane imaging was conducted with fluorescent secondary antibodies and an LI-COR Odyssey.

### 2.13 Statistical analysis

Statistical analysis of Western blot data was performed using the GraphPad Prism software. One-way ANOVA analysis followed by Dunnett's post test was applied to data sets. A *t*-test was used to compare the mean of 2 groups of data; *t*-tests were unpaired, 2 tailed with 95% confidence intervals.

## 3 Results

### 3.1 Exome Sequencing in familial ALS detects 16 protein-changing *TBK1* variants

We exome sequenced 699 index cases from a cohort of fALS from eleven countries, negative for mutations in all known ALS genes (including *SOD1*, *TDP43*, *C9ORF72*, *FUS*, *PFN1*, *UBQLN2*, *OPTN*, *VCP*, and *ANG*) and the intronic *C9ORF72* repeat expansion.

We identified 16 potentially deleterious protein-changing variants in *TBK1*, which were novel, or had an ExAC NFE carrier frequency of <1:20,000 individuals. A similar filtering strategy applied to the NFE subset of ExAC identified 54 variants from a total of 33,075 individuals, revealing a significant overabundance of protein-changing *TBK1* variants in our familiar cohort (*p*=1.02e^−10^, Fisher's 2-tailed test). Thirteen of these variants were absent from the following databases: 1000 genomes, UK10K, Exome Variant Server, and ExAC databases (n > 72,000). Two variants (p.R357Q, p.C471Y) were found once, and 1 variant (p.R358H) was found 7 times. Of the 16 variants identified, 4 were nonsense, 3 in-frame deletion, and 9 were missense variants (Table 1). The p.G217R variant is present in 2 Dutch cases predicted to be first-degree relatives (King kinship coefficient = 0.314, vcftools Ajk = 0.451) and is their only shared novel variant in a gene or pathway previously linked to ALS. The variant p.R357X is also present in a single FALS case in the ALS data browser (ALSdb); however, this is unlikely to be closely related to the p.R357X carrier identified in this study as they lack any other shared rare variants. Among the previously excluded FALS samples, another novel missense variant (p.Y394D) was identified in a patient who harbors the known pathogenic *TARDBP* mutation, p.M337V, which segregated in their affected sibling. However, the available exome data did not have sufficient coverage to determine if the sibling also shared the *TBK1* variant and DNA was not available to determine segregation by Sanger sequencing. Furthermore, the *TBK1* variant p.R358H was present in both first-degree relatives of a kindred, but both were also carriers for the particularly aggressive *FUS* p.R521C mutation.

**Table 1:** TBK1 mutations identified in ALS patients by this study and their clinical phenotype

Type of variant	Exon	Nucleotide variation^a^	Residue change	Number of cases	Control frequency	Gender	Clinical diagnosis	Site of onset	Age of onset (y)	Disease duration (mo)	Reference
Nonsense variants	2	c.4C>T	p.Gln2Ter	1	0/82,513	F	ALS	S	60	-	(Cirulli et al., 2015, Gijselinck et al., 2015)
	9	c.1069C>T	p.Arg357Ter	1{1}	0/82,519	F	ALS	UL	76	-	(Cirulli et al., 2015)
	18	c.1869_1875del	p.Met623IlefsdTer9	1	0/82,359	F	ALS	-	43	20.4	-
	18	c.1887_1890del	p.Gln629HisfsTer4	1	0/82,445	F	ALS	-	-	-	-
In-frame deletions	4	c.236_238delCAA	p.Thr79del	1	0/82,314	M	ALS	R	67.8	7.3	(van der Zee et al., 2017)
	-	992+1 G>A	p.Gly272_Thr331del	1{1}	0/80,635	M	ALS	-	46	24	-
	18	c.1928_1930delAAG	p.Glu643del	1{1}	0/82,323	M	ALS	UL	64	48	(Cirulli et al., 2015, Freischmidt et al., 2015, Gijselinck et al., 2015, van der Zee et al., 2017)
Missense variants	2	c.64A>C	p.Asn22His	1{1}	0/82,490	F	ALS	-	49	12	(Cirulli et al., 2015)
	2	c.92A>G	p.Thr31Ala	1	0/80,639	M	ALS	-	-	-	-
	5	c.385A>G	p.Asn129Asp	1	0/82,599	M	ALS	L	68	-	(Cirulli et al., 2015)
	6	c.649G>A	p.Gly217Arg	1 (1^b^)	0/82,595	M,M	ALS	-	-	-	(Cirulli et al., 2015)
	9	c.1070G>A	p.Arg357Gln	1 {1}	1/82,519	M	ALS	LL	50	94	(Freischmidt et al., 2015, Pozzi et al., 2017)
	9	c.1073G>A	p.Arg358His	1(1^b^)	7/82,519	-	-	-	-	-	-
	9	c.1180T>G	p.Tyr394Asp	1{1#}	0/82,080	F	ALS	B	40	-	(Cirulli et al., 2015)
	12	c.1412G>A	p.Cys471Tyr	1	1/79,400	F	ALS	-	-	-	(Cirulli et al., 2015)
	15	c.1694A>C	p.Gln565Pro	1 {1}	0/75,603	F	ALS	UL	50	36	(Cirulli et al., 2015)

The number in brackets ({}) represents the cases reported in ALSdb (http://alsdb.org/index.jsp).

{#} represents duplicated sample found in ALSdb.

Key: ALS, amyotrophic lateral sclerosis; B, bulbar; L, limb; LL, lower limb; R, respiratory; S, spinal; TBK1, TANK binding kinase 1; UL, upper limb.

a Mutation nomenclature as recommended by the Human Genome Variation Society www.hgvs.org, utilizing +1 as the A of the initiator Met codon, translation start site.

b Affected relative from the present cohort. Cases number = 699.

By mapping *TBK1* missense separately from nonsense variants, we observed difference in their distribution (Fig. 1A and B). While nonsense variants were spread across the whole protein, missense variants tended to cluster within the KD and ULD. Interestingly, the ULD has been reported to interact with both the SDD and the KD. These interactions play a vital role in the correct folding and dimerization of TBK1 (Li et al., 2012, Tu et al., 2013).

### 3.2 Selection of variants likely to be pathogenic

To focus our functional characterization of *TBK1* variants identified in our cohort, we concentrated on 3 variants. We firstly chose the missense p.G217R variant as 2 related individuals carried this mutation and only 1 *TBK1* missense mutation had previously been shown to segregate with disease (van der Zee et al., 2017). The second nonsense variant p.R357X was selected as it was present in ALSdb. Three variants (p.G217R, p.R357X, and p.C471Y) all scored highly using algorithms that predicted the variant to be damaging, and they appeared to be within functional domains according to the TBK1 homodimer crystal structure (PDB 4IM0) (Fig. 1C). The p.G217R mutation is located in the KD and was predicted to be damaging by 17/20 of applied algorithms. The p.R357X mutation is located in the ULD and found to remove the entire SDD. The p.C471Y is located in the SDD and may therefore impair TBK1 homodimerization (Fig. 1A and B).

### 3.3 ALS-linked *TBK1* variants decrease the phosphorylation of the TBK1 target IRF3

**Fig. 2:**
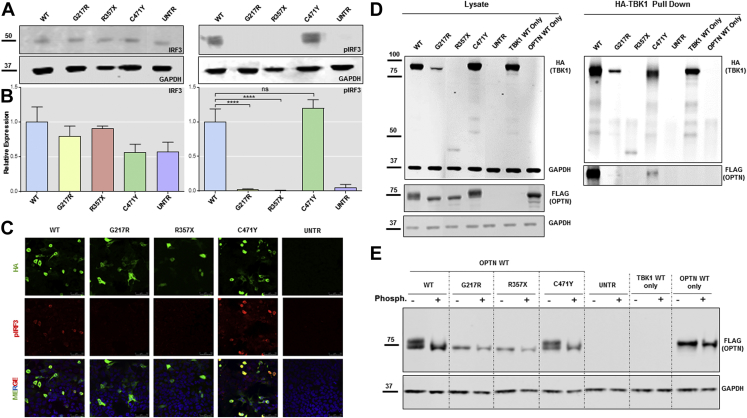
TBK1 p.G217R and p.R357X impair IRF3 phosphorylation as well as TBK1 binding with OPTN and its phosphorylation. (A) Western blot analysis of IRF3 (left) and pIRF3 (right). (B left) Quantitative analysis of blot in (A) left showing a similar expression level of endogenous IRF3 in cells (n = 3). (B right) Quantitative analysis of blot in (A) right showing a significant decrease of expression of endogenous pIRF3 in cells transfected with TBK1-p.G217R and p.R357X (n = 3, analyzed by one-way ANOVA followed by Dunnett's post test *p* < 0.0001). (C) Qualitative immunocytochemistry of HEK293T transfected with TBK1-WT, p.G217R, p.R357X, and probed for p-IRF3 confirming the result obtained by Western blot analysis (scale bar = 50 μm). (D) Co-IP with HA-tag pull down (TBK1) showing no binding of OPTN in any of the mutated samples with the exception of p.C471Y (n = 3). (E) HEK293T were transiently cotransfected with Flag-OPTN WT and HA-TBK1 WT, p.G217R, p.R357X, or p.C471Y, treated with alkaline phosphatase and analyzed by Western blot showing lack of OPTN phosphorylation in all mutated samples a part from p.C471Y. Abbreviations: IRF3, interferon regulatory factor 3; OPTN, optineurin; TBK1, TANK binding kinase 1; WT, wild type. **** *p* ≤ 0.0001.

Some ALS- and FTD-associated *TBK1* variants have previously been shown to diminish or abolish phosphorylation of the TBK1 target IRF3 (Freischmidt et al., 2015, Kim et al., 2016, Pozzi et al., 2017, Tsai et al., 2016). To test the efficiency of p.G217R, p.R357X, and p.C471Y on IRF3 phosphorylation, we transiently transfected HEK293T cells with WT or mutant *TBK1* and quantified IRF3 phosphorylation by Western blot. The expression levels of total IRF3 were comparable for WT and mutant constructs (Fig. 2A and B); however, levels of phospho-IRF3 (pIRF3) were significantly reduced in p.G217R and p.R357X variant compared with the WT by Western blot (Fig. 2A and B) and immunocytochemistry (Fig. 2D). Interestingly, the p.C471Y variant, predicted to be pathogenic by our bioinformatic tools, showed no difference from WT. Thus, both missense p.G217R and nonsense p.R357X mutations, but not the p.C471Y variant, abolished TBK1 kinase activity on its target IRF3.

### 3.4 ALS-linked *TBK1* variants decrease binding to OPTN and its phosphorylation

TBK1 is known to phosphorylate and regulate the activity of OPTN, a key receptor for polyubiquitinated proteins and mitochondria in autophagy pathways (Richter et al., 2016). TBK1 binds to the N-terminal region of OPTN (26–119), via its C-terminal domain (residues 677–729) (Li et al., 2016) and phosphorylates it on Ser177 (Wild et al., 2011) and Ser473 (Heo et al., 2015, Richter et al., 2016). Because mutations in OPTN are also linked to ALS, we tested whether our ALS-associated *TBK1* variants affected its ability to bind to OPTN and phosphorylate it. Co-IP of HA-tagged WT and p.C471Y TBK1 consistently pulled down flag-tagged OPTN (Fig. 2C); however, p.G217R and p.R357X dramatically reduced TBK1 binding to OPTN. This finding was validated by the observation that the same 2 mutants also failed to phosphorylate OPTN. Although TBK1 WT and p.C471Y generated a higher band on Western blot that disappeared in the presence of alkaline phosphatase, the higher band is absent following cotransfection with OPTN WT and TBK1 p.G217R or p.R357X (Fig. 2E). We conclude that both missense p.G217R and nonsense p.R357X mutations, but not p.C471Y, impair TBK1 binding to and phosphorylation of OPTN.

### 3.5 ALS-linked *TBK1* variants decrease the phosphorylation of TBK1

**Fig. 3:**
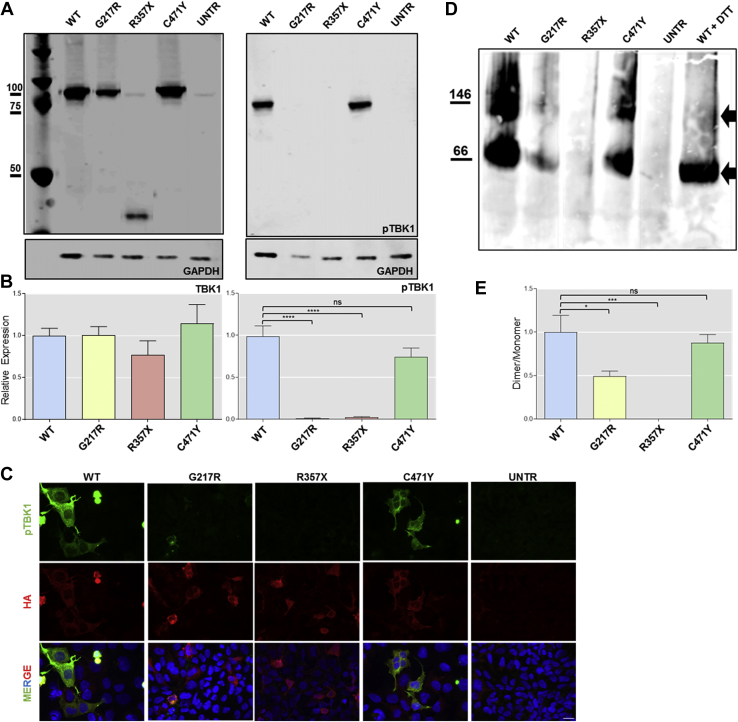
TBK1 p.G217R and p.R357X impair TBK1 phosphorylation and autophosphorylation and might reduce TBK1 homodimerization. (A) Western blot analysis of TBK1 expression levels (left) and pTBK1 (right). (B left) Quantitative analysis of blot in (A) left showing a similar expression level of TBK1 in cells (n = 4). (B right) Quantitative analysis of blot in (A) right showing a significant decrease of expression of pTBK1 in p.G217R and p.R357X (n = 4 analyzed by one-Way ANOVA followed by Dunnett's post test *p* < 0.0001). (C) Immunocytochemistry of HEK293T transfected with TBK1-WT, p.G217R, p.R357X, and probed for p-TBK1 confirming the result obtained by Western blot analysis (Fig. 3B) (Scale bar = 10 μm). (D) Native gel showing the dimer and monomer (indicated by black arrows) in TBK1-WT and TBK1-C471Y, the weaker dimer and monomer in the p.G217R sample, and no dimer or monomer in the R357X sample. (E) Quantitative analysis of gel in (D) showing significant reduction in dimer formation for p.G217R (positive control on the right TBK1 WT treated with DTT, n = 3, analyzed by one-way ANOVA followed by Dunnett's post test *p* < 0.05). Abbreviations: TBK1, tank binding kinase 1; WT, wild type. * *p* = 0.0306; *** *p* = 0.0006; **** *p* ≤ 0.0001.

TBK1 is activated by the phosphorylation of Ser172, which causes a critical change in protein conformation promoting the active position of the C-helix (Tu et al., 2013). We therefore tested whether the ALS-associated *TBK1* variants affect the phosphorylation and autophosphorylation of TBK1 itself. Western blots of HEK293T cells, transfected with each variant, were probed with an antibody specific for phospho-S172. Total TBK1 expression was similar for WT and all of the ALS-associated variants (Fig. 3A and C). Robust levels of phospho-S172-TBK1 were evident in WT and p.C471Y transfected cells but were absent in cells expressing p.G217R and p.R357X (Fig. 3A and B). Similarly, transfected HEK293T cells stained for phospho-S172-TBK1 by immunocytochemistry confirmed that TBK1 phosphorylation was absent in cells expressing p.G217R and p.R357X mutants (Fig. 3E). This indicates that the missense p.G217R and nonsense p.R357X but not the p.C471Y variant abolished the capacity of TBK1 for autophosphorylation.

### 3.6 ALS-linked variant p.G217R disrupts TBK1 homodimerization

TBK1 has to homodimerize to be functional and does so via a central axis formed by aligning the 2 SDD domains in a parallel orientation. The ULD and KD domains interact with each other at one end of the dimer creating a globular structure and stabilizing the homodimer (Tu et al., 2013) (Fig. 1C). We, therefore, investigated whether any of our variants affected the homodimerization of TBK1 by transfecting HEK293T cells with TBK1 WT, p.G217R, p.R357X, and p.C471Y and ran the lysates on nondenaturing gels (Fig. 3B). Native blots revealed 2 strong high– and low–molecular weight bands for WT and p.C471Y TBK1, indicating that a similar proportion exists as a homodimer and monomer. Little dimerization, however, was evident for the p.G217R mutant, and no band was visible for the p.R357X truncation mutant (Fig. 3B and D). Quantification of the dimer/monomer ratio confirmed that the missense p.G217R KD mutation showed a significantly lower level of TBK1 homodimerization compared with WT (*p* < 0.05) (Fig. 3D).

### 3.7 TBK1 phosphorylation in ALS patient LCLs

**Supplementary figure 1:**
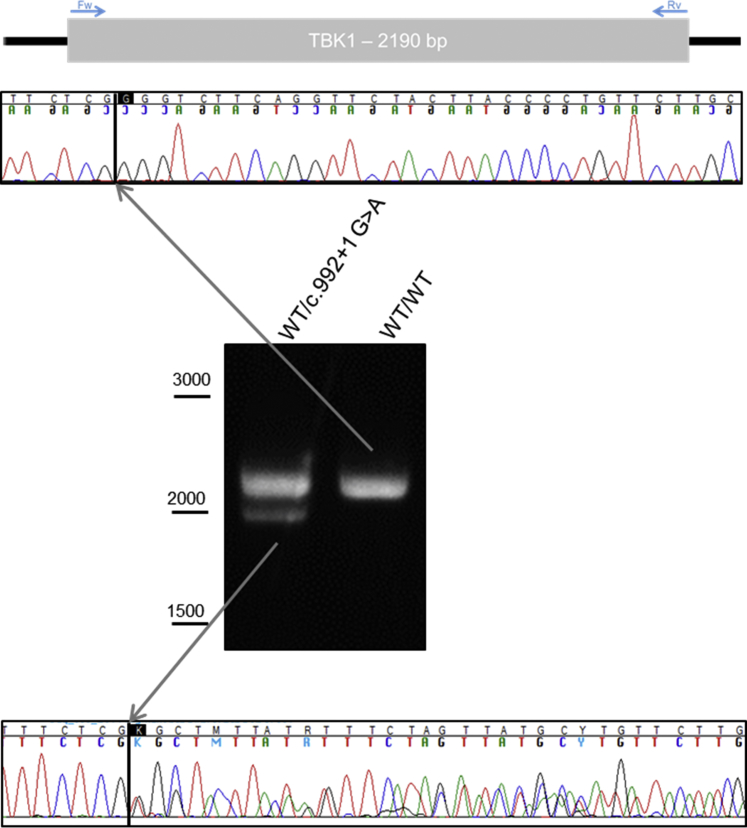
TBK1 variant c.992+1G>A produces the in-framedeletion of exon 8. Agarose gel on cDNA extracted from WT and c.992+1G<A (p.G272-T331 del) LCLs showing two different sized bands for the WT allele and the mutated allele carrying c.992+1G>A compared to the sample carrying both WT alleles (WT/C992+1G>A WTallele: 2190 bp, WT/C992+1G>A mutated allele: 1990bp, WT/WT: 2190bp). The boxes above and below show the sequencing of the WT/WT sample and the sequencing of the WT/c.992+1G>A sample, respectively.

To assess whether TBK1 activation is altered in ALS, we measured total TBK1 and phospho-TBK1 in patients' LCLs heterozygous for *TBK1* variants: p.T31A, c.992+1G>A (p.G272-T331del), p.R358H, p.Q565P, p.E643del, and 7 control LCLs. First, we sought to determine whether the c.992+1G>A causes an in-frame deletion of exon 8 within the ULD. cDNA was amplified by PCR from the patient-derived LCL harboring the c.992+1G>A variant using primers flanking the whole of TBK1 and run an agarose gel (Supplementary Fig. 1). We observed a lower band reflecting a ∼200 bp deletion, corresponding to the size of exon 8 and similar to the previously reported c.992+1G<T (Gijselinck et al., 2015) and (c.992+4_992+7delAGTA) mutations (van der Zee et al., 2017). The skipping of exon 8 was confirmed by Sanger sequencing.

**Fig. 4:**
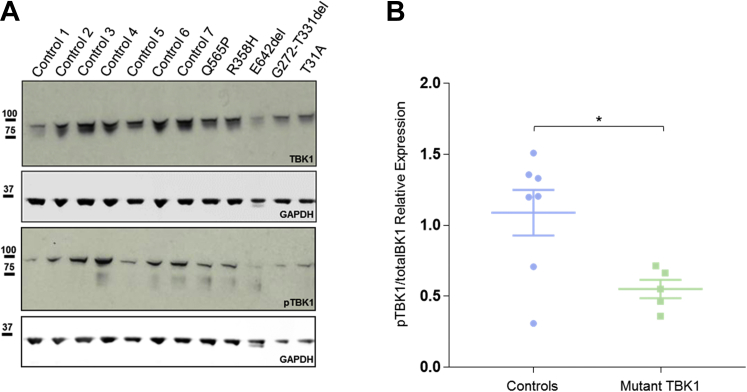
Patient-derived LCLs harboring *TBK1* variants show a reduced level of TBK1 phosphorylation. (A top) Western blot of control- and patient-derived LCLs, harboring 5 different TBK1 variants, showing the level of total TBK1. (A bottom) Western blot showing phospho-TBK1 expression in patient- and control-derived lymphoblasts. (B) Dot plot showing a significant difference in the ratio of phospho-TBK1 and total TBK1 between control- and patient-derived LCLs (analyzed by unpaired *t*-test, 2 tailed, *p* = 0.0229). Abbreviations: LCL, lymphoblastoid cell line; TBK1, tank binding kinase 1. * *p* = 0.0229.

Quantification of Western blots confirmed that total TBK1 was expressed at a similar level in all of the LCLs. Probing the same blots for phospho-S172-TBK1 revealed that the ratio between pTBK1 and total TBK1 is significantly different between patient- and control-derived LCLs (*p* = 0.0229, Fig. 4). Therefore, missense TBK1 mutations lead to reduced phosphorylation by self-interaction or with other kinases.

## 4 Discussion

In this study, we systematically analyzed samples from 699 index fALS patients and identified 16 *TBK1* variants including 4 nonsense mutations, predicted to cause haploinsufficiency by nonsense-mediated RNA decay or to be translated as truncated proteins. Three were in-frame deletions and 9 missense mutations, which appear to cluster in the functional kinase and ULDs known to play a role in TBK1 homodimerization (Li et al., 2012, Tu et al., 2013). Five of these variants have never been published before: p.M623fs, p.Q629fs, p.T31A, p.R358H, and c.992+1G>A (predicted to splice out the whole of exon 8 resulting in an in-frame deletion within the ULD). The c.992+1G>A variant was reported once in ALSdb. Of the other variants, only p.R357Q, p.E643del, and p.T79del have been functionally investigated (Freischmidt et al., 2015, Gijselinck et al., 2015, van der Zee et al., 2017). Interestingly, disease onset in the patient harboring both the *TBK1* p.Y394D and *TARDBP* p.M337V mutations was 40 years (Table 1), which is 20 years earlier than the average age of onset described in literature (Pozzi et al., 2017). This double-hit phenomenon was also described by Freischmidt et al. in the patients (all 3 affected relatives) harboring *TBK1* p.Y185X and *FUS* p.R524G (Freischmidt et al., 2015).

We characterized the functional impact of 3 ALS-linked variants selected on the basis of their predicted disruption to key functional domains within TBK1 (Fig. 1C). We chose p.G217R as it lies within the KD and was found in a putative affected sibling, p.R357X as it lies within the ULD, and was found in an unrelated case in ALSdb and p.C471Y, which lies within the SDD. Only p.G217R and p.R357X *TBK1* variants, but not p.C471Y, abolished the phosphorylation of IRF3. This has previously been described for other ALS-associated mutations (Freischmidt et al., 2015, Kim et al., 2016, Tsai et al., 2016). The same *TBK1* variants, p.G217R and p.R357X, also abolished TBK1 binding to OPTN and prevented its phosphorylation. The inhibition of TBK1 binding to OPTN has been previously observed in ALS-linked TBK1 variants, mainly located in the C-terminal region of the protein (Freischmidt et al., 2015, Kim et al., 2016, Pozzi et al., 2017, Tsai et al., 2016). However, the inhibition OPTN phosphorylation due to TBK1 mutations has never been shown before. A reduction in active OPTN phosphorylation would impair its function as a receptor for polyubiquitinated proteins and result in the accumulation of TDP-43, which has been observed in patients harboring *TBK1* mutations (Gijselinck et al., 2015, Pottier et al., 2015, van der Zee et al., 2017).

To become activated, TBK1 must form a homodimer and be phosphorylated either by itself or by other kinases (Tu et al., 2013). Here, we show that the p.G217R and p.R357X variants impair TBK1 autophosphorylation and its ability to be phosphorylated. This observation is consistent with a recent study that showed diminished TBK1 phosphorylation in ALS-associated *TBK1* in-frame deletions (p.T79del, p.D167del, p.E643del) (van der Zee et al., 2017). We have also shown that p.G217R, although located in the KD, affects TBK1 ability to homodimerize. In contrast, the p.C471Y variant within the SDD is able to phosphorylate and homodimerize at equivalent levels to the WT TBK1 protein and shows no evidence of pathogenicity.

Finally, we demonstrated that there is a significant difference between phospho-TBK1 and total TBK1 ratio in patient- compared with control-derived LCLs. This supports the hypothesis that disease-linked TBK1 variants might impair TBK1 autophosphorylation, disrupting its ability to bind and phosphorylate multiple partners including OPTN.

## 5 Conclusions

We have identified 4 novel and 12 previously described *TBK1* variants in ALS patients. Our functional studies demonstrated that the missense mutation p.G217R in the KD has an almost identical profile as the truncation p.R357X in the ULD and dramatically impairs the ability of TBK1 to form homodimers, autophosphorylate, and function as a kinase. Furthermore, the proportion of TBK1 that is activated is significantly reduced in 5 lymphoblast ALS patient lines carrying missense or in-frame deletion mutations. Thus, missense mutations in critical functional domains may cause disease through a loss of TBK1 function supporting functional haploinsufficiency as a common TBK1 disease mechanism (Cirulli et al., 2015, Freischmidt et al., 2015, Freischmidt et al., 2016). Further investigation of how ALS-linked *TBK1* variants alter TBK1 structure, phosphorylation, and dimerization will help unravel the disease pathogenesis and identify novel therapeutic targets.

## Disclosure statement

The authors have no actual or potential conflicts of interest.
